# Conservative versus surgical treatment for Garden I hip fracture: A randomized controlled trial protocol

**DOI:** 10.1097/MD.0000000000023378

**Published:** 2020-12-24

**Authors:** Wei Wang, Fangzhu Xu, Jianguang Luo, Liping Zhu

**Affiliations:** a Department of Orthopaedics, Zhenhai District People's Hospital of Ningbo, Zhejiang; b Department of Orthopaedics, the First People's Hospital of Jingzhou, Hubei, China.

**Keywords:** femoral neck fracture, hemiarthroplasty, non-displaced, protocol

## Abstract

**Background:**

A femoral neck fracture (FNF) is one of the most destructive and familiar injuries encountered via the orthopedic surgeons. However, this is no guideline for the treatment of the Garden I hip fractures because the current evidence is limited from the poor study design and small sample size. The objective of our research is to compare the safety and effectiveness of the surgical treatment and conservative treatment in the non-displaced FNFs.

**Methods:**

This is a randomized trial, which will be implemented from December 2020 to December 2021. The experiment was granted through the Research Ethics Committee of the Zhenhai District People's Hospital of Ningbo (2014005). Hundred patients meet inclusion criteria and exclusion criteria are included. Patients who are eligible for the following conditions will be included: those over 75 years old with Garden I hip fractures diagnosed by CT or X-ray. Patients with the following conditions will be excluded: patients age under 75 years old, the avascular necrosis of the femoral head, pathological fracture, infection, former symptomatic hip pathology, the history of hip fracture, as well as the lower limb deformity. The primary outcome contains pain at 1 month, 3 months and 6 months and hip function at 1 month, 3 months and 6 months. Secondary outcome includes the life quality, mortality rate, complications such as deep venous thrombosis, pulmonary embolism.

**Results:**

Comparison of outcome indicators in 2 groups after conservative treatment or surgical treatment (Table).

**Conclusion:**

The current trial will offer better evidence for the future treatment selection for Garden 1 FNFs for patients older than 75years old.

**Trial registration number:**

researchregistry6147.

## 1 Introduction

A femoral neck fracture (FNF) is one of the most destructive and familiar injuries encountered via the orthopedic surgeons.^[1,2]^ It is estimated that the number of patients worldwide will reach 63 million by 2050.^[3,4]^ According to reports, elderly patients account for the vast majority of the total number, particularly those over 80 years old. In the classification of Garden, Garden I hip fractures are expressed as the non-displaced FNFs, accounting for 15 to 20 percent of all the FNFs.^[5]^ The injury mechanism is the excessive external rotation leading to retroversion and valgus of femoral head. The blood supply of femoral head may be no loss or little, and owing to the impaction of 2 fragments, the fracture is stable.^[6,7]^

The choice of treatment is surgical or conservative. According to reports, surgical treatment is the best choice, due to the secondary displacement may cause damage to the blood supply of femoral head, and enhance the pressure in the joint capsule by generating the hematoma around fracture site, resulting in the delayed ischemic necrosis.^[8,9]^ Nevertheless, the complications of surgery are related to the increasing mortality and the socio-economic burden of the families and the medical systems. In some researches, patients receiving conservative treatment have achieved good results. Through the analysis of 54 patients with non-displaced FNFs, Helbig et al^[10]^ have found that 44% of patients did not have any complications in the process of conservative treatment, while 52% of patients needed surgical treatment owing to early fracture dislocation. There was no significant difference in patient satisfaction and survival rate between surgical treatment and conservative treatment. However, this is no guideline for the treatment of the Garden I hip fractures because the current evidence is limited from the poor study design and small sample size. The objective of our research is to compare the safety and effectiveness of the surgical treatment and conservative treatment in the non-displaced FNFs.

## 2 Materials and methods

The experiment will be implemented from December 2020 to December 2021 at the Zhenhai District People's Hospital of Ningbo. The experiment was granted through the Research Ethics Committee of the Zhenhai District People's Hospital of Ningbo (2014005) and recorded in research registry (researchregistry6147). The recruited patients are given the written informed consent before registration.

### 2.1 Inclusion criteria and exclusion criteria

Patients who are eligible for the following conditions will be included: those over 75 years old with Garden I hip fractures diagnosed by CT or X-ray. Patients with the following conditions will be excluded: patients age under 75 years old, the avascular necrosis of the femoral head, pathological fracture, infection, former symptomatic hip pathology, the history of hip fracture, as well as the lower limb deformity.

### 2.2 Randomization

Hundred patients meet inclusion criteria and exclusion criteria are included. In the random envelope, a random number is assigned to whole patients through the random-number table, and the distribution result is invisible. Patients are assigned randomly to conservative group (n = 50) and surgical group (n = 50).

### 2.3 Intervention

The non-surgical treatment contains early ambulation on the walking frame to reduce the load on affected side, and the tests of walking are carried out through a physical therapist with the medical supervision the day after the fracture. The follow-up radiographs are conducted at 1st, 3rd, and 6th week, involving the lateral and anteroposterior view of affected hip joint and the pelvis anteroposterior view. The patients are informed of the possibility of secondary displacement and the need for arthroplasty.

In the surgical groups, all the patients undergo the hemiarthroplasties in a lateral decubitus position through a modified hardinge approach. Prosthesis used is a cemented exeter stem and a bipolar head with 28 mm diameter inner head in all cases. Above processes used same cement using third-generation cementing techniques. After the surgery, all the patients receive 2 g of cefazolin for 3 days as an antibiotic prophylaxis, and low molecular weight heparin is subcutaneously injected ten days after the surgery to prevent thrombosis. In accordance with the standard post-operative rehabilitation, the physiotherapist offers the patients with mobility instructions, involving tolerable weight-bearing.

### 2.4 Outcomes

The primary outcome contains pain at 1 month, 3 months, and 6 months and hip function at 1 month, 3 months, and 6 months. Secondary outcome includes the life quality, mortality rate, complications such as deep venous thrombosis, pulmonary embolism.

### 2.5 Statistical analysis

Through utilizing the software of IBM SPSS Statistics for Windows, version 20, all the data can be analyzed (IBM Corp., Armonk, NY, USA). Afterwards, all the data are described with appropriate characteristics such as mean, median, standard deviation as well as percentage. The qualitative parameters for the groups are evaluated by *t* test. The categorical variables are determined by the χ^2^ tests. When *P* is less than .05, it is viewed to be significant in statistics.

## 3 Results

Comparison of outcome indicators in 2 groups after conservative treatment or surgical treatment will be shown in Table 1.

**Table 1:** Comparison of outcome indicators in 2 groups after conservative treatment or surgical treatment.

Surgical group (n = 50)	Conservative group (n = 50)	*P* level
Pain score		
1 month		
3 month		
6 month		
Harris hip score		
1 month		
3 month		
6 month		
Quality of life		
1 month		
3 month		
6 month		
Complications		
Deep venous thrombosis		
Pulmonary infection		
Pulmonary embolism		
Pressure ulcer		

## 4 Discussion

Hip fractures are the leading cause of morbidity, injury and mortality in the elderly patients.^[11,12]^ The number of FNFs is projected to increase rapidly due to the aging population. In the United States, more than 150,000 FNFs occur each year, which will double by 2050.^[13]^ Among FNFs, displaced fractures are more common, while one-thirds of total FNFs are non-displaced fractures. Based on the system of Garden classification, the non-displaced FNF can be classified on an anteroposterior X-ray.^[14]^

So far, there is no consensus on the best treatment of Garden I fractures. Taha et al^[15]^ have found that the conservative treatment only provided 44.3% of the non-displaced FNF healing rate. Nevertheless, Raaymakers et al^[16]^ found that the success rate of the conservative treatment was 85.9%. Surgical treatment also appears to be an excellent option, decreasing nonunion rates and secondary displacement. However, postoperative complications are the major concern especially for elderly patients.^[17,18]^ Non-operative treatment for Garden I FNFs is a rare utilized strategy, and its indications remain controversial. There is no convincing predictor of the outcome, partly because of the small number of the published studies. We implement this current protocol to assess the percentage of secondary displacement in Garden I FNFs, and to compare the safety and effectiveness of the surgical treatment and conservative treatment in the non-displaced FNF patients

## 5 Conclusion

The current trial will offer better evidence for the future treatment selection for Garden 1 FNFs for patients older than 75years old.

## Author contributions

Liping Zhu plans the study design. Jianguang Luo reviews the protocol. Fangzhu Xu collects data. Wei Wang write the manuscript. All authors approve the submission.

**Conceptualization:** Fangzhu Xu.

**Funding acquisition:** Liping Zhu.

**Investigation:** Fangzhu Xu.

**Software:** Jianguang Luo.

**Writing - original draft:** Wei Wang.
